# The Combination of Berberine, Tocotrienols and Coffee Extracts Improves Metabolic Profile and Liver Steatosis by the Modulation of Gut Microbiota and Hepatic miR-122 and miR-34a Expression in Mice

**DOI:** 10.3390/nu13041281

**Published:** 2021-04-13

**Authors:** Valentina Cossiga, Vincenzo Lembo, Cecilia Nigro, Paola Mirra, Claudia Miele, Valeria D’Argenio, Alessia Leone, Giovanna Mazzone, Iolanda Veneruso, Maria Guido, Francesco Beguinot, Nicola Caporaso, Filomena Morisco

**Affiliations:** 1 Gastroenterology Unit, Department of Clinical Medicine and Surgery, University of Naples ‘‘Federico II’’, 80131 Naples, Italy; valentina.cossiga@gmail.com (V.C.); v.lembo@hotmail.it (V.L.); giovanna.mazzone@gmail.com (G.M.); nicola.caporaso@unina.it (N.C.); 2 URT Genomics of Diabetes, Institute of Experimental Endocrinology and Oncology, National Research Council & Department of Translational Medical Sciences, University of Naples “Federico II”, 80131 Naples, Italy; c.nigro@ieos.cnr.it (C.N.); paolamirra06@gmail.com (P.M.); c.miele@ieos.cnr.it (C.M.); aleleone86@libero.it (A.L.); francesco.beguinot@unina.it (F.B.); 3 Department of Human Sciences and Quality of Life Promotion, San Raffaele Open University, Via di Val Cannuta 247, 00166 Rome, Italy; dargenio@ceinge.unina.it; 4 CEINGE-Biotecnologie Avanzate Scarl, Via Gaetano Salvatore 486, 80145 Napoli, Italy; venerusoiolanda95@gmail.com; 5 Task Force on Microbiome Studies, University of Naples “Federico II”, 80131 Naples, Italy; 6 Department of Molecular Medicine and Medical Biotechnologies, Federico II University, Via Sergio Pansini 5, 80131 Napoli, Italy; 7 Surgical Pathology & Citopathology Unit, Department of Medicine–DIMED, University of Padua, 35122 Padua, Italy; mguido@unipd.it

**Keywords:** insulin resistance, metabolic syndrome, miR-34a, miR-122, gut microbiome, NAFLD

## Abstract

Non-alcoholic-fatty liver disease (NAFLD) is spreading worldwide. Specific drugs for NAFLD are not yet available, even if some plant extracts show beneficial properties. We evaluated the effects of a combination, composed by *Berberis Aristata, Elaeis Guineensis* and *Coffea Canephora*, on the development of obesity, hepatic steatosis, insulin-resistance and on the modulation of hepatic microRNAs (miRNA) levels and microbiota composition in a mouse model of liver damage. C57BL/6 mice were fed with standard diet (SD, *n* = 8), high fat diet (HFD, *n* = 8) or HFD plus plant extracts (HFD+E, *n* = 8) for 24 weeks. Liver expression of miR-122 and miR-34a was evaluated by quantitativePCR. Microbiome analysis was performed on cecal content by 16S rRNA sequencing. HFD+E-mice showed lower body weight (*p* < 0.01), amelioration of insulin-sensitivity (*p* = 0.021), total cholesterol (*p* = 0.014), low-density-lipoprotein-cholesterol (*p* < 0.001), alanine-aminotransferase (*p* = 0.038) and hepatic steatosis compared to HFD-mice. While a decrease of hepatic miR-122 and increase of miR-34a were observed in HFD-mice compared to SD-mice, both these miRNAs had similar levels to SD-mice in HFD+E-mice. Moreover, a different microbial composition was found between SD- and HFD-mice, with a partial rescue of dysbiosis in HFD+E-mice. This combination of plant extracts had a beneficial effect on HFD-induced NAFLD by the modulation of miR-122, miR-34a and gut microbiome.

## 1. Introduction

In parallel to the global epidemic of obesity during the last decades, the increased prevalence of non-alcoholic fatty liver disease (NAFLD) has brought this pathology to the forefront of health care concerns [1,2]. Since it has a close association with obesity and insulin resistance, NAFLD has been described as the hepatic manifestation of the metabolic syndrome [3].

Although great efforts have been made to unravel the molecular mechanisms involved in this pathology [4], to date there are no approved drugs for the treatment of NAFLD and, therefore, lifestyle modification is the main therapeutic option for patients with liver steatosis [5]. However, some plant extracts are used as alternative nutraceutical approach on account of their well-recognized health benefits that may contribute to the improvement of NAFLD [6,7,8].

Previous in vitro and in vivo studies suggest that berberine, a plant alkaloid present in *Berberis Aristata*, increases the expression of low-density lipoprotein (LDL) receptors and, therefore, reduces the serum levels of LDL cholesterol [9,10]. Furthermore, berberine seems to be able to improve insulin resistance [11] and hepatic steatosis [12,13]. Several studies have also shown that tocotrienols, the less common form of vitamin E which are present in *Elaeis Guineensis*, reduce cholesterol and triglyceride levels through the modulation of lipogenic genes [14,15] and improve the antioxidant defense system of the cells [16]. Furthermore, experimental studies have shown that coffee also improves antioxidant status and reduces fat deposition in the liver through the gene expression modulation of the tumor necrosis factor-α (TNF-α), peroxisome proliferator-activated receptor-α (PPAR-α) and mitochondrial chaperones [17,18].

MicroRNAs (miRNAs) represent a class of non-coding RNA molecules consisting of approximately 22 nucleotides. They play an important role in the regulation of several crucial biological pathways by affecting gene expression at the post-transcriptional level [19]. Increasing evidence suggests that miRNAs are key epigenetic regulators of the lipid and glucose metabolism [20] and that alterations in their expression contribute to the development of liver steatosis [21,22,23].

In recent years, in the pathogenesis of NAFLD, the gut microbiota has emerged as a key regulator and as an actionable target for the development of specific therapies [24,25,26]. In presence of microbial dysbiosis and leaky gut, bacteria and their products trigger proinflammatory pathways which result in hepatocellular inflammation and fibrosis [27,28].

The aim of this study was to evaluate the effects of a combination of plant extracts, composed by *Berberis Aristata, Elaeis Guineensis* and decaffeinated green coffee from *Coffea Canephora* (Trixy^®^, Nathura S.p.A., Montecchio Emilia, RE, Italy), added to a high fat diet (HFD) in a mouse model of NAFLD. The effects of this extract combination on the development of obesity, hepatic steatosis and insulin resistance have been investigated. Moreover, the hepatic levels of specific miRNAs and the gut microbiota composition have been also studied.

## 2. Materials and Methods

### 2.1. Study Design and Dietary Intervention

Twenty-four male 4-week-old C57BL/6J mice were purchased from Envigo Italy (Milan, Italy). Mice were housed in standardized condition for animal facilities with a 12-h light/dark cycle, temperature conditions of 22 ± 1 °C. Food and water were provided ad libitum. After 1 week of acclimation the mice were divided into three groups (*n* = 8 each) and assigned into one of the following 24 weeks diets: (1) Standard diet (SD); (2) HFD; (3) HFD enriched with plant extracts (HFD+E) (140 mg/Kg/die) The HFD composition was 60% of energy derived from fats, 23% from proteins and 17% from carbohydrates, 5.6 kcal/g (Mucedola, Italy). The SD composition was 3% of energy derived from fats, 18.5% from proteins and 78.5% from carbohydrates, 3.3 kcal/g (Mucedola, Italy). A detailed composition of the diets is reported in Supplementary Table S1. The weight and food intake of mice were recorded weekly. At the end of the treatment, the mice were fasted overnight, and then euthanized by an overdose of avertin. Animal experiments were approved by the local ethics committee (Prot. *n*. 432/2016-PR) and the procedure were conducted within the animal welfare regulations and guidelines of the Italian National law D.L. 04/03/2014, *n*.26, about the use of animals for research.

### 2.2. Plant Extracts

A mixture of plant extracts in powder form, consisting of *Berberis Aristata, Elaeis Guineensis* and decaffeinated green coffee from *Coffea Canephora*, were added to diet preparation. The plant extracts were generously donated by Nathura S.p.A., Italy. The concentration of plant extracts was 140 mg/Kg of body weight per day, of which 103.3 mg of *Berberis Aristata* (87.84 mg of berberine), 25.1 mg of *Elaeis Guineensis* (5.27 mg of tocotrienols), and 11.8 mg of decaffeinated green coffee from *Coffea Canephora* (5.28 mg of chlorogenic acid). The dose of the extracts was chosen based on the effective quantities of the plant extracts adjusted to the body surface area of the mouse [29].

### 2.3. Insulin Tolerance Tests

At the end of diet protocol, mice were fasted for 4 h and then received an intraperitoneal injection of insulin (0.75 U/kg body weight) to test the insulin tolerance. Blood samples (5 μL) were collected from the tail vein before and at 15, 30, 45, 60, 90 and 120 min after the bolus of insulin. Blood glucose was measured using a portable glucometer (OneTouch Verio Flex System Kit, Johnson & Johnson Medical S.p.a., Pomezia, RM, Italy).

### 2.4. Liver Histology

At the time of sacrifice, mouse livers were fixed in 10% buffered formalin and embedded in paraffin. Liver tissue was cut into 4-μm-thick sections that were stained with hematoxylin and eosin. All slides were evaluated using a light microscope by an experienced pathologist who was blinded to the experiments. Specimens were scored for the severity of hepatocellular steatosis (percentage of affected hepatocytes), ballooning degeneration (present or absent), and necro-inflammation (present or absent). Steatosis was evaluated as percentage of affected hepatocytes on a 0> to 3 scale: 0 = absent ≤ 5%, 1 ≥ %–≤ 33%, 2 ≥ 33%–≤ 66%, 3 ≥ 66%.

### 2.5. Serum Measurements

At 24 weeks of diet, alanine aminotransferase (ALT), glucose, total cholesterol, low- and high-density lipoproteins (LDL and HDL) cholesterol serum levels were measured using a Modular Autoanalyzer (Pentra 400, HORIBA ABX, HORIBA Medical, Rome, Italy). Serum insulin was determined using an ELISA assay (Bio-Rad, Hercules, CA, USA) according to the manufacturer’s protocol.

### 2.6. RNA Isolation, Reverse Transcription, and Real-Time PCR

In order to investigate the molecular mechanisms underlying the improvements in hepatic steatosis and serum metabolic profiles due to the supplementation of HFD with the plant extracts, the hepatic levels of miR-122 and miR-34a have been analyzed in the three groups of mice. In this analysis, miRNA selection criteria are based on their well-documented role in or association with lipid metabolism, steatosis and NAFLD [30,31]. Disruption and homogenization of mouse liver samples were performed in *QIAzol* Lysis Reagent (QIAGEN, Hilden, Germany) using the TissueLyser LT (QIAGEN srl, Hilden, Germany). Total RNA was isolated from mouse liver samples by miRNeasy mini kit (QIAGEN srl, Hilden, Germany), according to the manufacturer’s instructions. After quantification by NanoDrop 2000 spectrophotometer (Thermo Scientific, Waltham, MA, USA), reverse transcription of 1 μg of total RNA was performed using the miScript II RT Kit (QIAGEN), according to the manufacturer’s instructions. Quantitative real-time PCR assays were performed in triplicate using miScript SYBR Green PCR Kit (QIAGEN) on iCycler Real-Time Detection System (Bio-Rad Laboratories, Hercules, CA, USA) and relative quantification of miRNA expression was calculated by the ΔΔCt method [32]. Each Ct value was normalized to the respective U6 snRNA Ct value of a sample to account for variability in the concentration of RNA and in the conversion efficiency of the RT reaction. Data were reported as arbitrary units (REU). Specific primers used for amplification were purchased from QIAGEN: Mm_miR-34a_1 miScript Primer Assay, MS00001428; Hs_miR-122a _1 miScript Primer Assay MS00003416; RNU6B_13 miScript Primer Assay, MS00014000.

### 2.7. Gut Microbiome Analysis

The cecal content was collected from each mouse at time of sacrifice, immediately cooled in dry ice and stored at −80 °C until time of the analysis. Genomic DNA was obtained from each collected stool sample. DNA extraction was performed using the 16 LEV Blood DNA kit and the Maxwell 16 instrument (both from Promega, Madison, WI, USA). In particular, 100 mg of each sample were treated with 400 μL of lysis buffer, v ortexed to completely homogenize it, and incubated at 95 °C in thermomixer for 5 min at 800 rpm. After a centrifugation step at 13,000 rpm for 5 min, 300 μL of supernatant/sample were transferred into a new tube, treated with 30 μL of proteinase K, vortexed and incubated at 56 °C in thermomixer for 20 min at 500 rpm. Then, the samples are added to the LEV cartridge to complete the extraction and eluted in 100 μL of elution buffer. Genomic DNAs concentration and quality were assessed by Nanodrop spectrophotometer (Thermo Fisher Scientific, Waltham, MA, USA). Next, all DNA samples were diluted to 2 ng/μL before to proceed with libraries preparation. The DNA libraries were obtained using the Microbiota Solution B reagents (Arrow Diagnostics, Genova, Italy), following manufacturer instructions [33]. This kit allows the amplification of the V3, V4 and V6 hypervariable regions of the bacterial 16S rDNA gene. The first step provides the amplification of the target regions. Once amplicon quality has been assessed on a 2% agarose gel, the PCR products are purified using the AMPure beads (Beckman Coulter, Brea, CA, USA) and used as template for a second step PCR required to add a specific index/sample. Indeed, the index is a short sequence of nucleotides used as a unique barcode specifically tagging each sample. At the end of the index PCR, the amplicons are assessed for quality (Tape Station, Agilent Technologies, Santa Clara, CA, USA), and beads purified (AMPure beads). The obtained libraries were quantified with the Qubit fluorometer (Thermo Fisher Scientific) and diluted to a final concentration of 10 nM. Next, 3 μL of different libraries can be pooled before sequencing. The sequencing reactions were carried out on the MiSeq instrument using a V2 Nano PE 250 × 2 flowcell (Illumina, San Diego, CA, USA) and loading the libraries pool to a final concentration of 6 pM with a 10% of PhiX.

The FASTQ files produced at the end of sequencing have been analyzed using the Microbat system software (SmartSeq, Novara, Italy). This tool, specifically conceived for microbiome sequences analysis, allows a quality assessment of the reads and the taxonomic assignment within each sample. Moreover, it allows to associate several samples into a group and to export the OTU table and the taxonomy table for further analyses. Indeed, these files were used to perform additional evaluations using the web-based tool Microbiome Analyst [34,35]. The latter allows to carry out diversity analysis, composition and comparative analyses, and to predict metabolic potentials. OTU annotation was performed using QIIME [36]. Data filtering was done to remove low quality or low abundance reads, setting a prevalence filter of 20%. Several tests were used to evaluate samples richness and/or evenness and the ANOVA test was done to highlight significant differences. Bray–Curtis dissimilarity index was used to evaluate beta diversity coupled with PERMANOVA test to assess the significance of samples grouping. Differential abundance analysis was performed using univariate statistical comparisons based on parametric test (*t*-test/ANOVA); *p*-values were adjusted using FDR method.

### 2.8. Statistical Analyses

Numeric data are presented as means ± SD (standard deviation). Statistical analysis has been performed, as appropriate, by one-way analysis of variance (ANOVA) with Tukey’s test correction for multiple comparisons between groups, using GraphPad Prism 7.00 (GraphPad Software, San Diego, CA, USA). Correlation analysis was calculated using Pearson’s correlation coefficient.

## 3. Results

### 3.1. Effect of Plant Extracts on Metabolic Profile of HFD Mice

**Figure 1:**
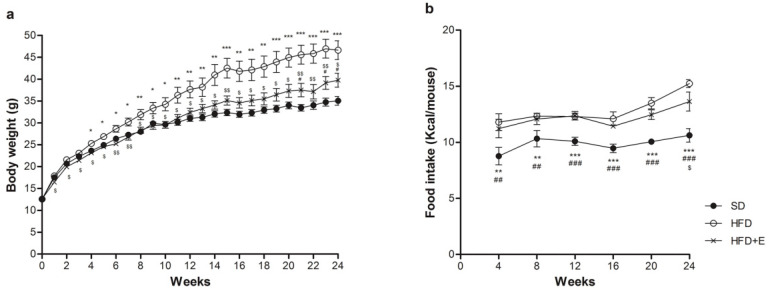
Effect of plant extracts on weight gain. Body weight was monitored every week (**a**), food intake every 4 weeks (**b**) during the diet protocol. SD: standard diet (black circles); HFD: high fat diet (white circles); HFD+E: high fat diet plus plant extracts (crosses); *n* = 8 per group. Data are presented as mean ± SD. * *p* < 0.05, ** *p* < 0.01, *** *p* < 0.001 HFD vs. SD, # *p* < 0.05, ## *p* < 0.01, ### *p* < 0.001 HFD+E vs. SD, $ *p* < 0.05, $$ *p* < 0.01 HFD vs. HFD+E.

All the C57BL/6J mice included in the study showed similar body weight at baseline (Figure 1a). During the diet protocol, HFD mice started to gain higher weight than SD mice already at the 4th week. HFD+E mice showed no significant difference in body weight compared to SD mice until the 21st week of diet, while maintained a lower body weight than HFD mice for the entire duration of diet protocol (Figure 1a). There were no differences in food intake between HFD and HFD+E mice until the 20th week of diet (Figure 1b). As expected, caloric intake was higher in both mice groups receiving HFD and HFD+E compared to SD mice (not shown).

To evaluate whether supplementation of plant extracts may have any effects on HFD-induced metabolic alteration, we analyzed metabolic parameters in the three groups of mice at 24 weeks of diet. Body weight was increased 1.37 fold in HFD and only 1.15 fold in HFD+E compared to SD. Serum fasting glucose was 2.3 and 2.2 fold increased in HFD and HFD+E mice, respectively, as compared to SD. Fasting insulinemia was 2.4-fold increased by HFD compared to SD, while no significant increase was detected in HFD+E compared to SD mice. Total cholesterol, LDL-cholesterol and LDL/HDL-cholesterol ratio are significantly increased by HFD, as expected, and reduced by 15%, 45% and 46%, respectively, by plant extracts supplementation to HFD (see Table 1).

**Figure 2:**
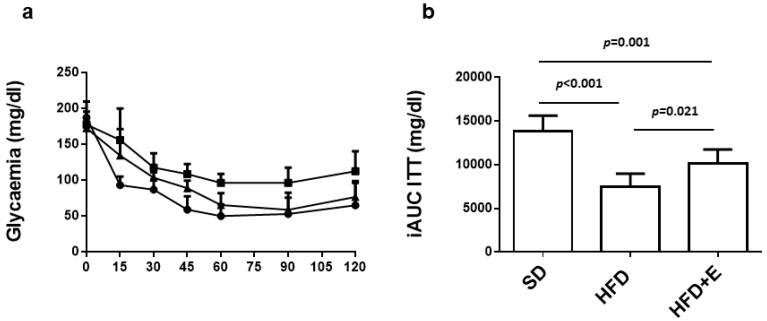
Effect of plant extracts on insulin sensitivity in HFD-fed mice. At 24 weeks of diet protocol, mice were subjected to insulin tolerance test. (**a**) Curves show the glycemic trend over 120 min following insulin bolus in SD (circles), HFD (squares) and HFD+E (triangles)-fed C57BL/6. (**b**) Bars show the inverse calculation of the area under the curves (iAUC) of the three groups of mice. Values are expressed as means ± SD of determinations in 8 mice per group. Statistical significance was evaluated using one-way ANOVA.

Following the insulin tolerance test (Figure 2a), glucose levels remained higher in HFD mice compared to SD mice during the entire length of the test. At variance, insulin sensitivity was improved in HFD+E group, as shown by a significant decrease of their blood glucose levels (Figure 2a). The iAUC indicates that HFD mice developed insulin resistance (7529 ± 1469 mg/dL) compared to SD mice (13,804 ± 1816 mg/dL) and, interestingly, HFD+E administration was able to induce a partial rescue of insulin sensitivity in mice (10,138 ± 1620 mg/dL) (Figure 2b).

### 3.2. Effect of Plant Extracts on Steatosis Development of HFD Mice

**Figure 3:**
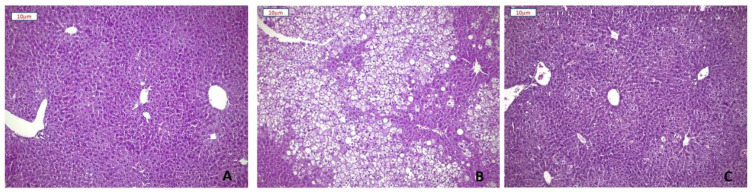
Effect of plant extracts on the development of hepatic steatosis. Representative images of Hematoxylin-Eosin staining of liver sections (10× magnification) from SD (**A**), HFD (**B**), and HFD+E mice (**C**).

Hematoxylin and eosin staining of liver sections showed severe (>66%) mixed micro-macrovescicular steatosis in all mice on HFD. In seven out of eight animals, steatosis involved more than 80% of hepatocytes, only sparing a rim of peri-portal cells. Steatosis was absent or minimal (<5%) in five out of eight mice receiving the plant extracts. SD mice showed normal liver tissue (Figure 3). Moreover, liver damage was reflected by the 2.8-fold increase of ALT levels in HFD-fed mice. Any significant increase in ALT levels was not revealed in HFD+E-fed mice (Table 1).

### 3.3. Effect of Plant Extracts on miR-122 and miR-34a Levels in HFD Mice

**Figure 4:**
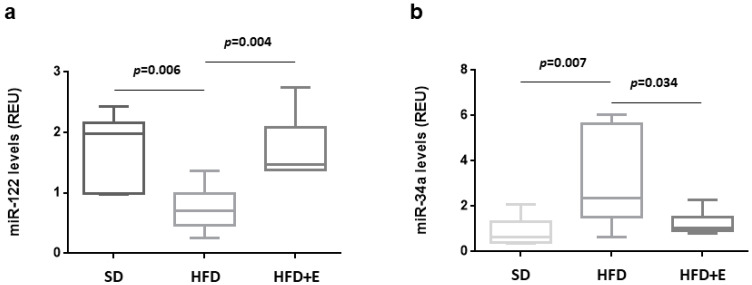
HFD and plant extracts effect on hepatic miRNAs expression. The expression of miR-122 (**a**) and miR-34a (**b**) was evaluated in hepatic tissue from SD, HFD and HFD+E-fed C57BL/6 mice by qRT-PCR. Data are shown as expression units relative to U6 snRNA levels and reported in box plots, where center lines show the medians, “+” symbols the means, box limits the 25th and 75th percentiles and whiskers extend 1.5 times the interquartile range from the 25th and 75th percentiles. Statistical significance was evaluated using one-way ANOVA.

In the present study, in comparison to SD, HFD induces a 50% decrease of miR-122 levels, which were completely rescued by the plant extracts co-administration (Figure 4a). Conversely, a four-fold increase of miR-34a was induced by HFD and its expression is reduced to levels similar to SD mice in mice fed with HFD+E diet (Figure 4b).

**Figure 5:**
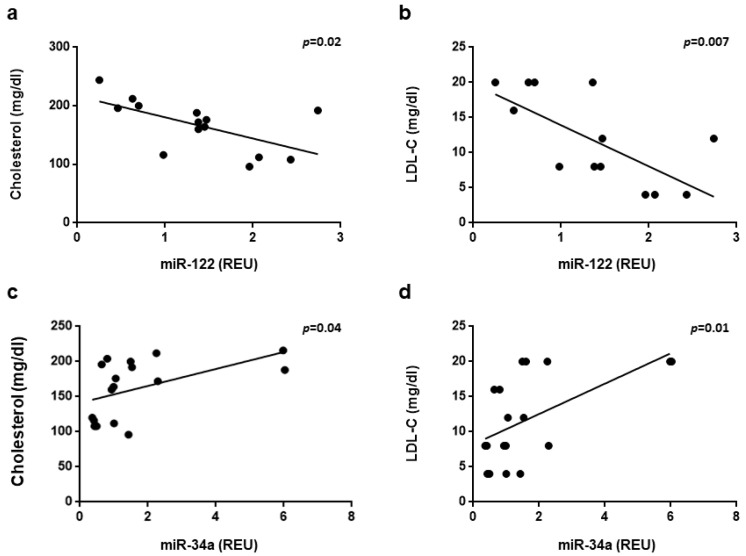
Correlation of hepatic miRNAs expression and metabolic parameters. Correlation of miR-122 (**a**,**b**) and miR-34a (**c**,**d**) expression with total cholesterol (**a**,**c**) and LDL-cholesterol (**b**,**d**) was assessed by linear regression analysis and calculated using Pearson’s correlation coefficient. Correlations with a *p* value < 0.05 was considered statistically significant.

A correlation analysis was performed between the expression levels of miR-122 and miR-34a in the livers of the three groups of mice and serum metabolic parameters. A significant negative correlation with total cholesterol and LDL-cholesterol was observed for miR-122 (r = 0.605, *p* = 0.02; r = 0.68, *p* = 0.007 respectively) (Figure 5a,b) Conversely, a significant positive correlation with total cholesterol and LDL-cholesterol was observed for miR-34a (r = 0.499, *p* = 0.0005; r = 0.573, *p* = 0.01 respectively) (Figure 5c,d).

### 3.4. Gut Microbiota Modifications

**Figure 6:**
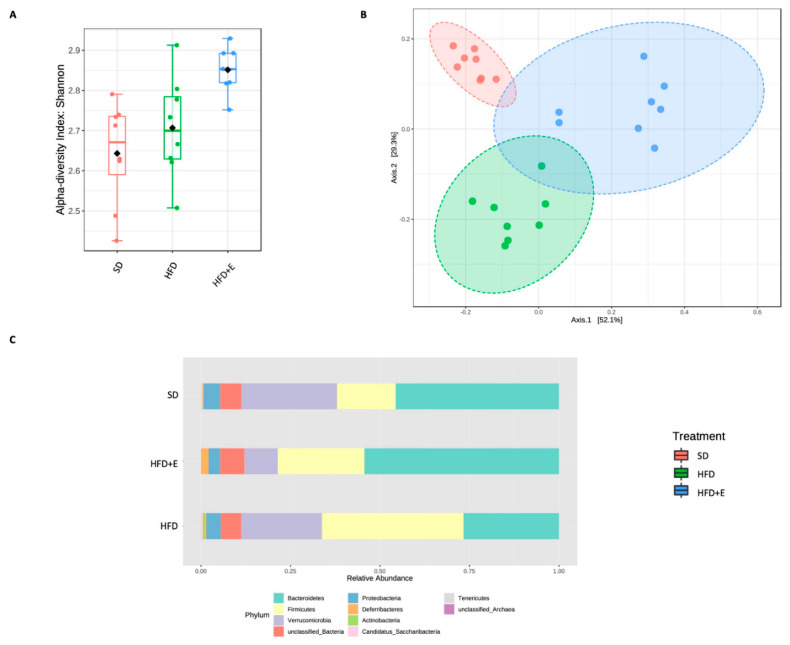
Bacterial communities profiling between SD, HFD and HFD+E treated mice. Alpha diversity was measured to assess any difference in the structure of bacterial communities among the study groups. Several metrics have been used to test the within-group diversity, Shannon index showing a significant difference (*p* = 0.003, ANOVA) between the HFD+E group and the others (**A**). To verify differences in the microbial composition between the three study groups, beta diversity has been also evaluated by Bray–Curtis metric. Statistical significance (*p* < 0.001) of grouping was assessed by ANOSIM (**B**). Taxonomic assignment highlights a different microbial composition between the three groups. Phyla variation into three groups (**C**).

The bacterial gut microbiome composition was evaluated in the three differentially fed mice-groups as described under Methods. Two sequencing runs were totally performed by analyzing 12 samples/run and obtaining an average of 40,324 reads/sample. Alpha diversity analysis was carried out to assess the within-group diversity using different metrics. Chao1 index and observed species number did not highlight any significant difference between the three groups. Moreover, richness of the gut microbiota was similar between SD and HFD groups. Thus, it is suggested that the HFD diet did not affect the bacterial richness. A not significant richness reduction was present in the HFD+E mice. Although no significant richness was observed by Chao1 and observed species indices, higher bacterial richness and evenness in the HFD+E mice compared to the other two groups was reported using the Shannon metric (*p* = 0.003) (Figure 6A).

Next, beta diversity was analyzed to verify the differences between samples; principal coordinate analysis (PCoA) showed a significant clusterization among the three groups (*p* < 0.001), suggesting their clear separation based on bacterial communities’ composition (Figure 6B). Abundances comparison showed a different taxa composition among the study groups (Figure 6C). Interestingly, at the phylum level, SD mice showed a higher abundance of Bacteroidetes and a lower abundance of Firmicutes compared to HFD mice. Moreover, this imbalance seems to be partially restored in the HFD+E group showing also the reduction of the Verrucomicrobia phylum.

**Figure 7:**
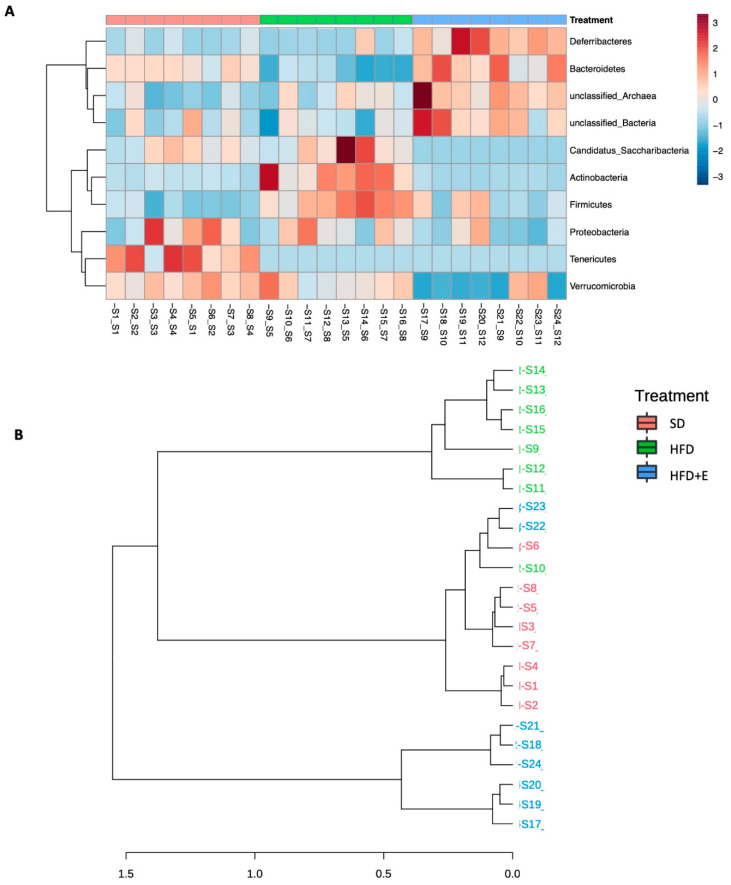
Microbial communities cluster analyses. Heatmap of variance was obtained by grouping the reads according to the observed taxa to evaluate abundance patterns. A clear cluster was obtained between the three groups at phylum level (**A**). This separation is observed also in the dendrogram analyses (**B**).

Thus, a clustering analysis was performed to visualize abundance patterns able to cluster the samples. As shown in Figure 7A, samples belonging to the same group were more similar than samples from the other groups at phylum level, and this behavior was present at all taxonomic levels (data not shown). This trend was confirmed also by dendrogram analysis showing a higher distance of HFD+E samples compared to the other two groups (Figure 7B). As shown in Figure 7A, lower Bacteroides and higher Firmicutes phyla were observed in HFD compared to SD group, but the HFD+E mice showed a partial correction of dysbiosis.

**Figure 8:**
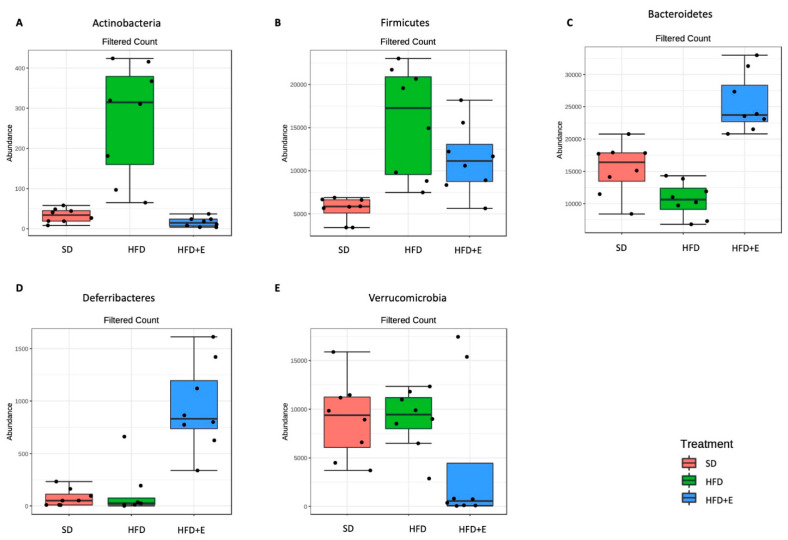
Significantly different taxa identified at phylum level between SD, HFD and HFD+E treated mice. Differential abundance analyses allowed to identify significantly different taxa between the three study groups. *t*-Test/ANOVA were used to assess significant results (adjusted *p*-value < 0.05, specific *p*-values are detailed in Supplementary Table S2). At phylum level, Actinobacteria (**A**) and Firmicutes (**B**) phyla are highly represented in the HFD mice respect to the other two groups. On the contrary, the Bacteroidetes phylum (**C**) is less abundant in the HFD than in the SD and in the HFD+E mice. Interestingly, the Deferribacteres (**D**) and the Verrucomicrobia (**E**) phyla are respectively high and low expressed in the HFD+E mice respect to both SD and HFD.

Finally, to highlight significant differences between the three differently fed mice groups, differential abundance analysis was performed. All the significantly expressed taxa from phylum to species level are reported in Supplementary Table S1. At phylum level, nine taxa are significantly different. Among these, the Actinobacteria (Figure 8A) and the Firmicutes (Figure 8B) phyla are most abundant in the HFD mice than in the other two groups, while Bacteroidetes is more abundant in the SD and, mostly, in the HFD+E groups than in the HFD mice (Figure 8C); finally, the Deferribacteres (Figure 8D) and the Verrucomicrobia (Figure 8E) phyla were respectively high and lower expressed in the HFD+E mice.

**Figure 9:**
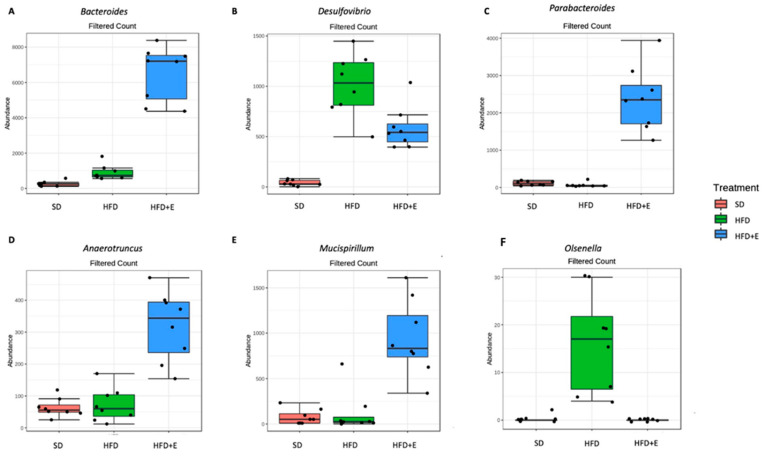
Significantly different taxa identified at genus level between SD, HFD and HFD+E treated mice. Significantly different taxa have been identified using differential abundance analyses coupled with *t*-test/ANOVA (adjusted *p*-value < 0.05). At genus level, the *Bacteroides* (**A**) was highly represented in the HFD+E treated mice than in the other two groups, as well as *Parabacteroides* (**C**), *Anaerotruncus* (**D**) and *Mucispirillum* (**E**) genera. On the other side, the genera Desulfovibrio (**B**) and Olsenella (**F**) were more abundant in the HFD than in the other groups.

At genus level, we found 54 differentially expressed taxa (Supplementary Table S2), the most significantly different being the *Bacteroides* genus that appear more abundant in the HFD+E mice than in the other two groups (Figure 9A).

## 4. Discussion

NAFLD is the most common cause of liver disease and, in association with metabolic comorbidities, it represents a worldwide health problem [37]. Currently, there are no licensed drugs approved for the treatment of hepatic steatosis and, therefore, lifestyle modification is the standard therapeutic approach recommended [5].

Our study demonstrates that the addition of the combination of plant extracts composed by *Berberis Aristata, Elaeis Guineensis* and *Coffea Canephora* to HFD induces the amelioration of metabolic parameters and of hepatic steatosis in mice, modulating miRNAs and gut microbiota.

Previous studies have analyzed either a single plant extract or the main bioactive compounds purified from plant extracts, documenting their therapeutic effect on the modulation of the glucose and lipid metabolism in diabetic or dyslipidemic patients, as well as in animal models [9,10,11,12,13,14,15,16,17,18]. Nonetheless, their combined effects and their mechanisms of action remain largely unexplored. In particular, berberine showed hypoglycemic, insulin-sensitizing and lipid-lowering effects through several mechanisms. Cameron J. et al. [9] showed that berberine increases the LDL receptor expression through the inhibition of PCSK9 (proprotein convertase subtilisin/kexin type 9). In addition, Zhang H. et al. demonstrated that berberine has its insulin-sensitizing effect through the activation of adenosine monophosphate-activated protein kinase (AMPK), which may play a role in reducing insulin resistance [12]. In relation to tocotrienols effects, Burdeos GC et al. [15] showed that its lipid-lowering effects occurs through the modulation of lipogenic gene expression, such as Srbf1 (sterol regulatory element-binding transcription factor 1). Finally, Vitaglione P. et al. [18] showed that the addition of decaffeinated coffee to a high fat diet (HFD) in rats determined a reduction in hepatic fat accumulation, systemic and liver oxidative stress, and liver inflammation.

Studies indicate that mice fed with a fatty diet develop insulin resistance and hepatic steatosis [38]. Insulin resistance plays a key role in the development of hepatic steatosis by increasing both free fatty acid delivery from the adipose tissue and de novo lipogenesis in the liver [39,40]. Our results show that C57BL/6J mice fed with an HFD develop obesity associated with insulin resistance. Interestingly, the administration of plant extracts reduces the weight gain and the metabolic alterations during the HFD period, although the quantity of food intake was the same in the two different groups. The lower body weight in the HFD+E mice may contribute to the higher insulin sensitivity compared to the HFD mice at the end of the diet period. The data shown here demonstrate that HFD+E mice have lower fasting insulin levels than HFD mice. The reduction of fasting insulinemia is consistent with the partial rescue of insulin tolerance, measured by ITT, in HFD+E mice compared to HFD mice. Although these data indicate that HFD+E improves insulin sensitivity, fasting glycaemia is similar in both HFD and HFD+E mice. In light of our data, we cannot exclude that tissue-specific alterations (such as higher basal hepatic glucose production) may contribute to keep high fasting glucose levels in HFD+E mice.

An ameliorative effect, due to the extracts’ administration, on the metabolic alterations induced by the HFD is also the reduction of total cholesterol levels and the ratio of LDL/HDL cholesterol found in the HFD+E mice compared with the HFD mice. Furthermore, histological analysis demonstrates that all the mice fed with HFD develop a severe hepatic steatosis, while the addition of the extracts reduces the accumulation of fat in the liver.

Recently, the causal role of miRNAs in the development and progression of NAFLD has been increasingly recognized [22]. MiR-122 is one of the most abundant miRNAs in the liver and several studies have demonstrated the hepatic downregulation of miR-122 in participants with fatty liver disease [41,42,43]. Moreover, the significance of miR-122 in the regulation of lipid metabolism and its part in the development of NAFLD has been strongly suggested by a number of studies both in vitro and in vivo [44,45,46,47]. In a steatotic hepatocyte model, human hepatic cell line L02 treated with oleic acid, the effect of miR-122 in the fat deposition has been observed [44] and the reduction of Yin Yang 1 (YY1) mRNA stability with the consequent upregulation of the Farnesoid X Receptor (FXR) signaling has been demonstrated as a contribution of miR-122 to the lipid droplet formation and hepatic triglyceride accumulation [45]. In addition, the silencing of miR-122 in another hepatocyte model HepG2 cells produces an expression pattern of key hepatic lipogenic genes similar to human NASH, including the mature Sterol Regulatory Element-Binding Transcription Factor isoform 1c and 2 (SREBP-1c and SREBP2) and their downstream targets Fatty Acid Synthase (FAS) and 3-Hydroxy-3-Methyl-Glutaryl-CoA reductase (HMGR) [46]. Using an in vivo loss-of-function model mice with germline knockout (KO) or liver-specific knockout (LKO) of the Mir122 locus, the prolonged absence of miR-122 leads to an accumulation of hepatic triglycerides in young mice. This abnormality is associated with the upregulation of several gene products that catalyze triglyceride biosynthesis and storage, including the direct miR-122 targets 1-Acylglycerol-3-Phosphate O-Acyltransferase 1 (AGPAT1) and Cell Death-Inducing DFFA-like Effector C (CIDEC), also known as Fat Specific Protein 27 (FSP27). Besides, the hepatic steatosis observed in miR-122a–deficient mice is also a consequence of the global impairment of lipid metabolism and lipoprotein assembly and secretion, as demonstrated by the repression of multiple genes involved in lipid metabolism [47,48]. Finally, miR-122 also regulates Hypoxia-Inducible Factor-1 (HIF-1 alpha) and Vimentin in hepatocytes, thus correlating with fibrosis in diet-induced steatohepatitis [49].

Besides miR-122, miR-34a is weakly expressed in the hepatocytes but it tightly regulates the lipid metabolism. In human hepatic cells and in mouse models of steatosis, miR-34a is able to regulate several genes, whose downregulation is associated with higher TG accumulation and liver steatosis. This is the case of Hepatocyte Nuclear Factor 4 α (HNF4α) [50], PPARα [51] and sirtuin 1 (SIRT1) [52,53]. miR-34a is also significantly upregulated in the liver of both animal models and human patients with NAFLD [54,55,56,57]. Our data have shown that the hepatic levels of miR-122 are reduced in the mice fed with an HFD, but they were comparable to those in the SD group when the plant extracts were added to the HFD. Conversely, increased hepatic levels of miR-34a are observed in the HFD group while lower levels are found in the HFD+E group, similarly to SD mice group. Therefore, our study has demonstrated that *Berberis Aristata, Elaeis Guineensis* and *Coffea Canephora* extracts administrated in combination are able to prevent alterations in the hepatic expression of both miR-122 and miR-34a caused by an HFD.

Furthermore, in the present study, the hepatic levels of miR-122 are inversely correlated with the serum levels of total cholesterol and LDL-cholesterol and those of miR-34a are directly correlated with the serum levels of total cholesterol and LDL-cholesterol, supporting the role of these miRNAs in the regulation of cholesterol and the fatty acid metabolism in the liver.

In an attempt to investigate the mechanisms underlying the beneficial effects of this extract combination, the analysis of the gut microbiome has gained our attention, since: i. a large number of recent studies have showed that herbal medicines are capable of reversing the abnormal gut microbiota composition in diseased human cohorts and model animals [58,59], and ii. gut-microbiota derived metabolites are capable to regulate miRNA expression with effects on metabolism [60,61]. 16S rRNA sequencing analysis was carried out to assess the gut microbial composition of HFD compared to SD fed mice and verify the effect of plant extracts addiction to HFD in the modulation of the gut microbiota composition. First, we were able to assess a different bacterial composition in the gut microbiota of SD and HFD mice. Moreover, the HFD+E mice showed its own microbiota suggesting that the plant extracts are able to modulate the microbiota promoting the establishment of specific bacteria. As already reported elsewhere [62,63], the HFD mice had a significant increased abundance of Firmicutes and a decreased abundance of Bacteroidetes respect to SD. This alteration is restored in the HFD+E treatment group, supporting a beneficial effect of the treatment.

In the present study, changes in 54 taxa, even genus level, were reported. In particular, the genus *Bacteroides*, belonging to the Bacteroidetes phylum, was the most significantly different and was highly abundant in the HFD+E mice. The phylum Bacteroidetes is known to be involved in short chain fatty acids (SCFAs) production in the gut, starting from dietary fibers [63]. It has been assessed that SCFAs, moving into the blood circulation, are able to exert positive effects on lipid metabolism in the liver [64]. Thus, this may be a mechanism through which the plant extracts, by modulating the bacterial dysbiosis at gut level, may exert a beneficial effect on liver functions. On the contrary, the genus *Desulfovibrio* was highly represented in the HFD mice respect to the other groups (Figure 9B). *Desulfovibrio* is a sulphate-reducing bacterium that has been found increased in association with inflammatory bowel diseases [65]. As a consequence, its abundant reduction in the HFD+E mice may ameliorate some NAFLD-related clinical features. Furthermore, it is interesting to underline that some species were highly abundant in the HFD+E treated group. In particular, we found an increased abundance of the genera *Parabacteroides* (Figure 9C), *Anaerotruncus* (Figure 9D), and *Mucispirillum* (Figure 9E).

A reduction of the genus *Parabacteroides* has been recently reported in neonatal cholestasis disease and it seems to exert a positive modulation on bile acids metabolism and obesity [66]. The *Anaerotruncus* genus has been found to negatively correlate with obesity in child [67] and to positively impact on the outcome of fecal transplantation in inflammatory bowel disease patients [68]. Finally, the genus *Mucispirillum* has been reported to exert a positive effect on gut inflammation [69].

On the other side, the genus *Olsenella* was significantly increased in the HFD group respect to the others (Figure 9F). This genus has been already reported as increased in the caecum of HFD models and it has been suggested it may be involved in gut permeability modifications.

Moreover, we found both in SD and HFD+E mice lower levels of the *Lachonospiraceae* family, present also at genus level (*unclassified_ Lachonospiraceae*) compared to the HFD group. A member of this family, the *Lachnoclostridium*, has been associated to colorectal cancer [70] and recently proposed to be related also to NAFLD [62].

This study has some limitations. Firstly, this is a pilot small animal study evaluating the combination of plant extracts on hepatic steatosis and more studies are needed to confirm these results. Secondly, the role of this combination on the modulation of miRNAs and gut microbiota must be clarified by further studies.

Conversely, the strength of our study is the use of the combination of three plant extracts, never previously evaluated, as novel therapeutic strategy for hepatic steatosis. Although it is not possible to discriminate which component of the extract mixture has led to the improvement of the metabolic profile, it is possible to hypothesize a synergistic action of the three plant components.

## 5. Conclusions

In conclusion, our study shows, for the first time, that this combination of plant extracts has a protective effect on obesity, hepatic steatosis, insulin resistance and dyslipidemia. The metabolic outcomes are associated with the expression of miR-122 and miR-34a in the liver. Although the role of miR-122 and miR-34a on the lipid metabolism and NAFLD has previously been highlighted, further studies will be useful to demonstrate how these miRNAs are modulated by these plant extracts in combination. The positive effect on the gut microbiota demonstrated here using the extract combination may be directly responsible for the miRNA expression in the liver, as previously published. However, we cannot exclude that the extract effects on miRNA hepatic levels and metabolism may be also indirectly dependent on gut microbiota, in light of the absence of weight gain. However, either way, the outcome of this work suggests the use of the combination of *Berberis Aristata, Elaeis Guineensis* and *Coffea Canephora* as an adjuvant therapy for the prevention of obesity-induced NAFLD, supporting the gut microbiota-miRNA pathway as a reasonable target for the therapy of NAFLD.

## Figures and Tables

**Table 1:** Metabolic parameters analyzed at 24 weeks of SD (standard diet), HFD (high fat diet) and HFD+E (high fat diet plus plant extracts) fed mice (*n* = 8 for each group).

Variable	SD	HFD	HFD+E	*p*-Value
Body weight (g)	34.6 ± 2.8	47.4 ± 6.0	40.0 ± 3.6	*p* = 0.001 (HFD vs. SD) *p* = 0.024 (HFD+E vs. SD) *p* = 0.018 (HFD+E vs. HFD)
Glycaemia (mg/dL)	85.6 ± 2.7	200.0 ± 41.3	191.1 ± 34.25	*p* < 0.001 (HFD vs. SD) *p* < 0.001 (HFD+E vs. SD)
Insulinemia (pg/mL)	473.9 ± 80.0	1156.3 ± 307.4	725.8 ± 270.0	*p* < 0.001 (HFD vs. SD) *p* = 0.046 (HFD+E vs. HFD)
Cholesterol (mg/dL)	110.0 ± 8.3	209.3 ± 19.9	178.0 ± 16.9	*p* < 0.001 (HFD vs. SD) *p* < 0.001 (HFD+E vs. SD) *p* = 0.014 (HFD+E vs. HFD)
LDL-Cholesterol (mg/dL)	5.3 ± 2.0	19.3 ± 1.6	10.7 ± 3.3	*p* < 0.001 (HFD vs. SD) *p* = 0.007 (HFD+E vs. SD) *p* < 0.001 (HFD+E vs. HFD)
LDL/HDL-Cholesterol	0.09 ± 0.03	0.28 ± 0.05	0.15 ± 0.05	*p* < 0.001 (HFD vs. SD) *p =* 0.024 (HFD+E vs. SD) *p* < 0.001 (HFD+E vs. HFD)
ALT (U/L)	29.3 ± 11.2	81.3 ± 47.1	38.7 ± 24.35	*p* < 0.025 (HFD vs. SD) *p* < 0.038 (HFD+E vs. HFD)

Data are presented as mean ± SD. Statistical significance was evaluated using one-way ANOVA.

## Data Availability

Data are available at University of Naples Federico II.

## Supplementary Materials

The following are available online at https://www.mdpi.com/article/10.3390/nu13041281/s1. Supplementary Table S1. Ingredients and composition of study diets for male C57BL/6J mice fed an SD or HDF alone or supplemented with a powder mixture of plant extracts consisting of *Berberis Aristata*, *Elaeis Guineensis* and decaffeinated green coffee from *Coffea Canephora* for 24 weeks. Supplementary Table S2. Full list of significantly different taxa identified by differential abundance analyses and assessed by T-Test/ANOVA and considering an adjusted *p*-value < 0.05 as threshold. Taxa are reported from phylum to species and ordered based on *p*-values (from the most significant value).

## References

[1] Andronescu C.I., Purcarea M.R., Babes P.A. (2018). Nonalcoholic fatty liver disease: Epidemiology, pathogenesis and therapeutic implications. J. Med. Life.

[2] Finck B.N. (2018). Targeting Metabolism, Insulin Resistance, and Diabetes to Treat Nonalcoholic Steatohepatitis. Diabetes.

[3] Katsiki N., Perez-Martinez P., Anagnostis P., Mikhailidis D.P., Karagiannis A. (2018). Is Nonalcoholic Fatty Liver Disease Indeed the Hepatic Manifestation of Metabolic Syndrome?. Curr. Vasc. Pharmacol..

[4] Marchisello S., Di Pino A., Scicali R., Urbano F., Piro S., Purrello F., Rabuazzo A.M. (2019). Pathophysiological, Molecular and Therapeutic Issues of Nonalcoholic Fatty Liver Disease: An Overview. Int. J. Mol. Sci..

[5] Bril F., Cusi K. (2017). Management of Nonalcoholic Fatty Liver Disease in Patients with Type 2 Diabetes: A Call to Action. Diabetes Care.

[6] Salomone F., Barbagallo I., Godos J., Lembo V., Currenti W., Cinà D., Avola R., D’Orazio N., Morisco F., Galvano F. (2017). Silibinin Restores NAD⁺ Levels and Induces the SIRT1/AMPK Pathway in Non-Alcoholic FattyLiver. Nutrients.

[7] Maithilikarpagaselvi N., Sridhar M.G., Swaminathan R.P., Sripradha R., Badhe B. (2016). Curcumin inhibits hyperlipidemia and hepatic fat accumulation in high-fructose-fed male Wistar rats. Pharm. Biol..

[8] Shang J., Chen L.L., Xiao F.X., Sun H., Ding H.C., Xiao H. (2008). Resveratrol improves non-alcoholic fatty liver disease by activating AMP-activated protein kinase. Acta Pharmacol. Sin..

[9] Cameron J., Ranheim T., Kulseth M.A., Leren T.P., Berge K.E. (2008). Berberine decreases PCSK9 expression in HepG2 cells. Atherosclerosis.

[10] Xiao H.B., Sun Z.L., Zhang H.B., Zhang D.S. (2012). Berberine inhibits dyslipidemia in C57BL/6 mice with lipopolysaccharide induced inflammation. Pharmacol. Rep..

[11] Kong W.J., Zhang H., Song D.Q., Xue R., Zhao W., Wei J., Wang Y.M., Shan N., Zhou Z.X., Yang P. (2009). Berberine reduces insulin resistance through protein kinase C-dependent up-regulation of insulin receptor expression. Metabolism.

[12] Zhang Z., Li B., Meng X., Yao S., Jin L., Yang J., Wang J., Zhang H., Zhang Z., Cai D. (2016). Berberine prevents progression from hepatic steatosis to steatohepatitis and fibrosis by reducing endoplasmic reticulum stress. Sci. Rep..

[13] Guo T., Woo S.L., Guo X., Li H., Zheng J., Botchlett R., Liu M., Pei Y., Xu H., Cai Y. (2016). Berberine Ameliorates Hepatic Steatosis and Suppresses Liver and Adipose Tissue Inflammation in Mice with Diet-induced Obesity. Sci. Rep..

[14] Song B.L., De Bose-Boyd R.A. (2006). Insig-dependent ubiquitination and degradation of 3-hydroxy-3-methylglutaryl coenzyme a reductase stimulated by delta- and gamma-tocotrienols. J. Biol. Chem..

[15] Burdeos G.C., Nakagawa K., Watanabe A., Kimura F., Miyazawa T. (2013). γ-Tocotrienol attenuates triglyceride through effect on lipogenic gene expressions in mouse hepatocellular carcinoma Hepa 1-6. J. Nutr. Sci. Vitaminol..

[16] Ahsan H., Ahad A., Iqbal J., Siddiqui W.A. (2014). Pharmacological potential of tocotrienols: A review. Nutr. Metab..

[17] Salomone F., Li Volti G., Vitaglione P., Morisco F., Fogliano V., Zappalà A., Palmigiano A., Garozzo D., Caporaso N., D’Argenio G. (2014). Coffee enhances the expression of chaperones and antioxidant proteins in rats with nonalcoholic fatty liver disease. Transl. Res..

[18] Vitaglione P., Morisco F., Mazzone G., Amoruso D.C., Ribecco M.T., Romano A., Fogliano V., Caporaso N., D’Argenio G. (2010). Coffee reduces liver damage in a rat model of steatohepatitis: The underlying mechanisms and the role of polyphenols and melanoidins. Hepatology.

[19] Hammond S.M. (2015). An overview of microRNAs. Adv. Drug Deliv. Rev..

[20] Suksangrat T., Phannasil P., Jitrapakdee S. (2019). miRNA Regulation of Glucose and Lipid Metabolism in Relation to Diabetes and Non-alcoholic Fatty Liver Disease. Adv. Exp. Med. Biol..

[21] Baffy G. (2015). MicroRNAs in Nonalcoholic Fatty Liver Disease. J. Clin. Med..

[22] Gjorgjieva M., Sobolewski C., Dolicka D., de Sousa M.C., Foti M. (2019). miRNAs and NAFLD: From pathophysiology to therapy. Gut.

[23] Sulaiman S.A., Muhsin N.I.A., Jamal R. (2019). Regulatory Non-coding RNAs Network in Non-alcoholic Fatty Liver Disease. Front. Physiol..

[24] Marra F., Svegliati-Baroni G. (2018). Lipotoxicity and the gut-liver axis in NASH pathogenesis. J. Hepatol..

[25] Betrapally N.S., Gillevet P.M., Bajaj J.S. (2016). Changes in the intestinal microbiome and alcoholic and nonalcoholic liver diseases: Causes or effects?. Gastroenterology.

[26] Putignani L., Alisi A., Nobili V. (2016). Pediatric NAFLD: The future role of patient-tailored probiotics therapy. J. Pediatr. Gastroenterol. Nutr..

[27] Rahman K., Desai C., Iyer S.S., Thorn N.E., Kumar P., Liu Y., Smith T., Neish A.S., Li H., Tan S. (2016). Loss of junctional adhesion molecule a promotes severe steatohepatitis in mice on a diet high in saturated fat, fructose, and cholesterol. Gastroenterology.

[28] Makri E., Goulas A., Polyzos S.A. (2020). Epidemiology, Pathogenesis, Diagnosis and Emerging Treatment of Nonalcoholic Fatty Liver Disease. Arch. Med. Res..

[29] Reagan-Shaw S., Nihal M., Ahmad N. (2008). Dose translation from animal to human studies revisited. Fed. Am. Soc. Exp. Biol. J..

[30] Liu W., Cao H., Yan J., Huang R., Ying H. (2015). ‘Micro-managers’ of hepatic lipid metabolism and NAFLD. Wiley Interdiscip. Rev. RNA.

[31] Rottiers V.L., Näär A.M. (2012). MicroRNAs in metabolism and metabolic disorders. Nat. Rev. Mol. Cell Biol..

[32] Livak K.J., Schmittgen T.D. (2001). Analysis of relative gene expression data using real time quantitative PCR and the 2(−ΔΔC(T)) Method. Methods.

[33] Carosso A., Revelli A., Gennarelli G., Canosa S., Cosma S., Borella F., Tancredi A., Paschero C., Boatti L., Zanotto E. (2020). Controlled ovarian stimulation and progesterone supplementation affect vaginal and endometrial microbiota in IVF cycles: A pilot study. J. Assist. Reprod. Genet..

[34] Chong J., Liu P., Zhou G., Xia J. (2020). Using MicrobiomeAnalyst for comprehensive statistical, functional, and meta-analysis of microbiome data. Nat. Protoc..

[35] Dhariwal A., Chong J., Habib S., King I., Agellon L.B., Xia J. (2017). MicrobiomeAnalyst—A web-based tool for comprehensive statistical, visual and meta-analysis of microbiome data. Nucleic Acids Res..

[36] Caporaso J.G., Kuczynski J., Stombaugh J., Bittinger K., Bushman F.D., Costello E.K., Fierer N., Pena A.G., Goodrich J.K., Gordon J.I. (2010). QIIME allows analysis of high-throughput community sequencing data. Nat. Methods.

[37] Younossi Z., Anstee Q.M., Marietti M., Hardy T., Henry L., Eslam M., George J., Bugianesi E. (2018). Global burden of NAFLD and NASH: Trends, predictions, risk factors and prevention. Nat. Rev. Gastroenterol. Hepatol..

[38] Kim S., Choi Y., Choi S., Choi Y., Park T. (2014). Dietary camphene attenuates hepatic steatosis and insulin resistance in mice. Obesity.

[39] Utzschneider K.M., Kahn S.E. (2006). Review: The role of insulin resistance in nonalcoholic fatty liver disease. J. Clin. Endocrinol. Metab..

[40] Tamura S., Shimomura I. (2005). Contribution of adipose tissue and de novo adipogenesis to nonalcoholic fatty liver disease. J. Clin. Investig..

[41] Latorre J., Moreno-Navarrete J.M., Mercader J.M., Sabater M., Rovira O., Gironès J., Ricart W., Fernandez-Real J.M., Ortega F.J. (2017). Decreased lipid metabolism but increased FA biosynthesis are coupled with changes in liver microRNAs in obese subjects with NAFLD. Int. J. Obes..

[42] Auguet T., Aragonès G., Berlanga A., Guiu-Jurado E., Marti A., Martinez S., Sabench F., Hernandéz M., Aguilar C., Sirvent J.J. (2016). miR33a/miR33b* and miR122 as Possible Contributors to Hepatic Lipid Metabolism in Obese Women with Nonalcoholic Fatty Liver Disease. Int. J. Mol. Sci..

[43] Braza-Boïls A., Marí-Alexandre J., Molina P., Arnau M.A., Barcelò-Molina M., Domingo D., Girbes J., Giner J., Martinez-Dolz L., Zorio E. (2016). Deregulated hepatic microRNAs underlie the association between non-alcoholic fatty liver disease and coronary artery disease. Liver Int..

[44] Nie Y.Q., Cao J., Zhou Y.J., Liang X., Du Y.L., Wan Y.J., Li Y.Y. (2014). The effect of miRNA-122 in regulating fat deposition in a cell line model. J. Cell Biochem..

[45] Wu G.Y., Rui C., Chen J.Q., Sho E., Zhan S.S., Yuan X.W., Ding Y.T. (2017). MicroRNA-122 Inhibits Lipid Droplet Formation and Hepatic Triglyceride Accumulation via Yin Yang. Cell. Physiol. Biochem..

[46] Cheung O., Puri P., Eicken C., Contos M.J., Mirshahi F., Maher J.W., Kellum J.M., Min H., Luketic V.A., Sanyal A.J. (2008). Nonalcoholic steatohepatitis is associated with altered hepatic MicroRNA expression. Hepatology.

[47] Hsu S., Wang B., Kota J., Yu J., Costinean S., Kutay H., Yu L., Bai S., La Perle K., Chivukula R.R. (2012). Essential metabolic, anti-inflammatory, and anti-tumorigenic functions of miR-122 in liver. J. Clin. Investig..

[48] Tsai W., Hsu S., Hsu C., Lai T., Chen S., Shen R., Huang Y., Chen H., Lee C., Tsai T. (2012). MicroRNA-122 plays a critical role in liver homeostasis and hepatocarcinogenesis. J. Clin. Investig..

[49] Csak T., Bala S., Lippai D., Satishchandran A., Catalano D., Koyds K., Szabo G. (2015). microRNA-122 regulates hypoxia-inducible factor-1 and vimentin in hepatocytes and correlates with fibrosis in diet-induced steatohepatitis. Liver Int..

[50] Xu Y., Zalzala M., Xu J., Li Y., Yin L., Zhang Y. (2015). A metabolic stress-inducible miR-34a-HNF4α pathway regulates lipid and lipoprotein metabolism. Nat. Commun..

[51] Ding J., Li M., Wan X., Jin X., Chen S., Yu C., Li Y. (2015). Effect of miR-34a in regulating steatosis by targeting PPARα expression in nonalcoholic fatty liver disease. Sci. Rep..

[52] Shan W., Gao L., Zeng W., Hu Y., Wang G., Li M., Zhou J., Ma X., Tian X., Yao J. (2015). Activation of the SIRT1/p66shc antiapoptosis pathway via carnosic acid-induced inhibition of miR-34a protects rats against nonalcoholic fatty liver disease. Cell. Death Dis..

[53] Castro R.E., Ferreira D.M., Afonso M.B., Borralho P.M., Machado M.V., Cortez-Pinto H., Rodrigues C.M.P. (2013). miR-34a/SIRT1/p53 is suppressed by ursodeoxycholic acid in the rat liver and activated by disease severity in human non-alcoholic fatty liver disease. J. Hepatol..

[54] Li S., Chen X., Zhang H., Liang X., Xiang Y., Yu C., Zen K., Li Y., Zhang C.Y. (2009). Differential expression of microRNAs in mouse liver under aberrant energy metabolic status. J. Lipid Res..

[55] Liu X.L., Pan Q., Zhang R.N., Shen F., Yan S.Y., Sun C., Xu Z.J., Chen Y.W., Fan J.G. (2016). Disease-specific miR-34a as diagnostic marker of non-alcoholic steatohepatitis in a Chinese population. World J. Gastroenterol..

[56] Salvoza N.C., Klinzing D.C., Gopez-Cervantes J., Baclig M.O. (2016). Association of Circulating Serum miR-34a and miR-122 with Dyslipidemia among Patients with Non-Alcoholic Fatty Liver Disease. PLoS ONE.

[57] Yamada H., Suzuki K., Ichino N., Ando Y., Sawada A., Osakabe K., Sugimoto K., Ohashi K., Teradaira R., Inoue T. (2013). Associations between circulating microRNAs (miR-21, miR-34a, miR-122 and miR-451) and non-alcoholic fatty liver. Clin. Chim. Acta.

[58] Zhang R., Gao X., Bai H., Ning K. (2020). Traditional Chinese Medicine and Gut Microbiome: Their Respective and Concert Effects on Healthcare. Front. Pharmacol..

[59] Chung E., Elmassry M.M., Kottapalli P., Kaur G., Dufour J.M., Wright K., Ramalingam L., Moustaid-Moussa N., Wang R., Hamood A.N. (2020). Metabolic benefits of annatto-extracted tocotrienol on glucose homeostasis, inflammation, and gut microbiome. Nutr. Res..

[60] Virtue A.T., McCright S.J., Wright J.M., Jimenez M.T., Mowel W.K., Kotzin J.J., Joannas L., Basavappa M.G., Spencer S.P., Clark M.L. (2019). The gut microbiota regulates white adipose tissue inflammation and obesity via a family of microRNAs. Sci. Transl. Med..

[61] Jia N., Lin X., Ma S., Ge S., Mu S., Yang C., Shi S., Gao L., Xu J., Bo T. (2018). Amelioration of hepatic steatosis is associated with modulation of gut microbiota and suppression of hepatic miR-34a in *Gynostemma pentaphylla* (Thunb.) Makino treated mice. Nutr. Metab..

[62] Cui H., Li Y., Wang Y., Jin L., Yang L., Wang L., Liao J., Wang H., Peng Y., Zhang Z. (2020). Da-Chai-Hu Decoction Ameliorates High Fat Diet-Induced Nonalcoholic Fatty Liver Disease Through Remodeling the Gut Microbiota and Modulating the Serum Metabolism. Front. Pharmacol..

[63] Li J., Hu Q., Xiao-Yu D., Zhu L., Miao Y.F., Kang H.X., Zhao X.L., Yao J.Q., Long D., Tang W.F. (2020). Effect of Sheng-Jiang Powder on Gut Microbiota in High-Fat Diet-Induced NAFLD. Evid. Based Complement. Alternat. Med..

[64] Nazli A., Yang P.C., Jury J., Howe K., Watson J.L., Soderholm J.D., Sherman P.M., Perdue M.H., McKay D.M. (2004). Epithelia under metabolic stress perceive commensal bacteria as a threat. Am. J. Pathol..

[65] Chen Y.R., Zhou L.Z., Fang S.T., Long H.Y., Chen J.Y., Zhang G.X. (2019). Isolation of Desulfovibrio spp. from human gut microbiota using a next-generation sequencing directed culture method. Lett. Appl. Microbiol..

[66] Li M., Liu S., Wang M., Hu H., Yin J., Liu C., Huang Y. (2020). Gut Microbiota Dysbiosis Associated with Bile Acid Metabolism in Neonatal Cholestasis Disease. Sci. Rep..

[67] Hou Y.P., He Q., Ouyang H.M., Peng H.S., Wang Q., Li J., Lv X.F., Zheng Y.N., Li S.C., Liu H.L. (2017). Human Gut Microbiota Associated with Obesity in Chinese Children and Adolescents. Biomed. Res. Int..

[68] Satokari R., Fuentes S., Mattila E., Jalanka J., de Vos W.M., Arkkila P. (2014). Fecal transplantation treatment of antibiotic-induced, noninfectious colitis and long-term microbiota follow-up. Case Rep. Med..

[69] Herp S., Brugiroux S., Garzetti D., Ring D., Jochum L.M., Beutler M., Eberl C., Hussain S., Walter S., Gerlach R.G. (2019). Mucispirillum schaedleri antagonizes Salmonella virulence to protect mice against colitis. Cell Host Microbe.

[70] Liang J.Q., Li T., Nakatsu G., Chen Y.X., Yu J. (2019). A novel faecal lachnoclostridium marker for the non-invasive diagnosis of colorectal adenoma and cancer. Gut.

