# Supplementary figures and images for: The Combination of Berberine, Tocotrienols and Coffee Extracts Improves Metabolic Profile and Liver Steatosis by the Modulation of Gut Microbiota and Hepatic miR-122 and miR-34a Expression in Mice
Source: Nutrients. 2021 Apr 13;13(4):1281. doi: 10.3390/nu13041281 (PMC8069822; doi:10.3390/nu13041281)

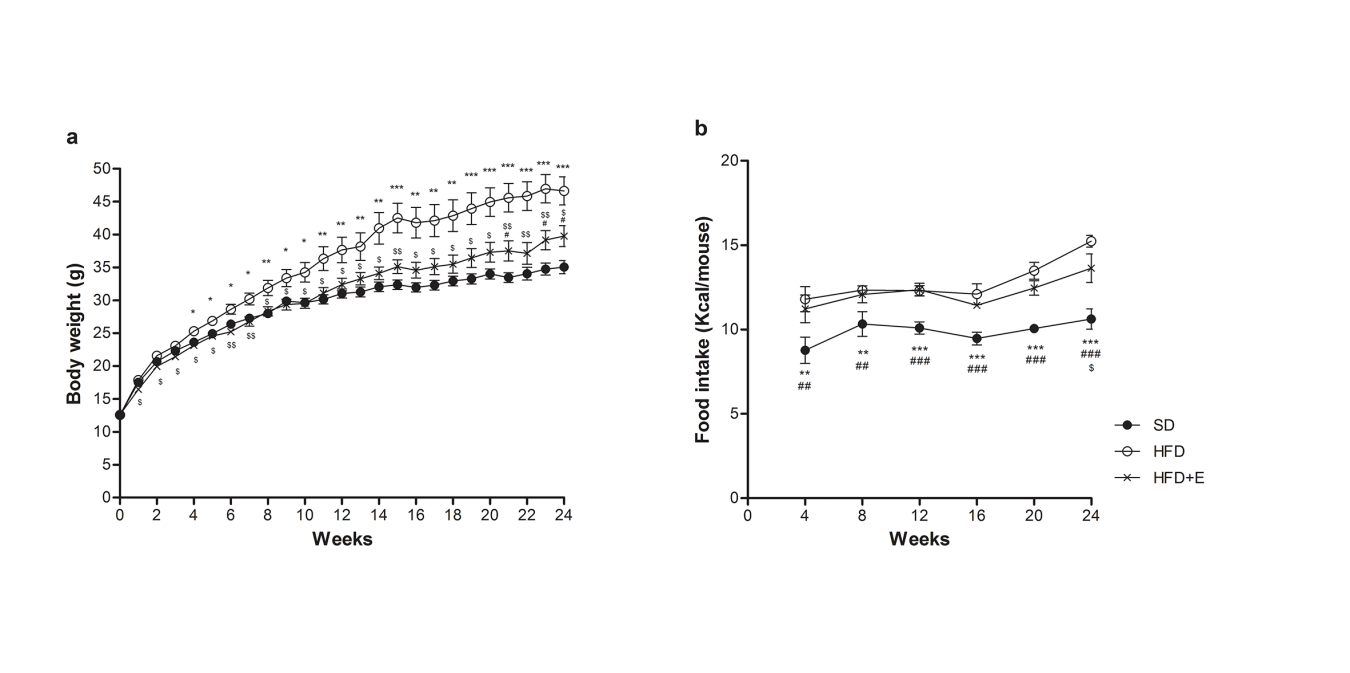

Supplement: Supplementary file 1 [file nutrients-13-01281-s001.zip › nutrients figures/Figure 1.tif]

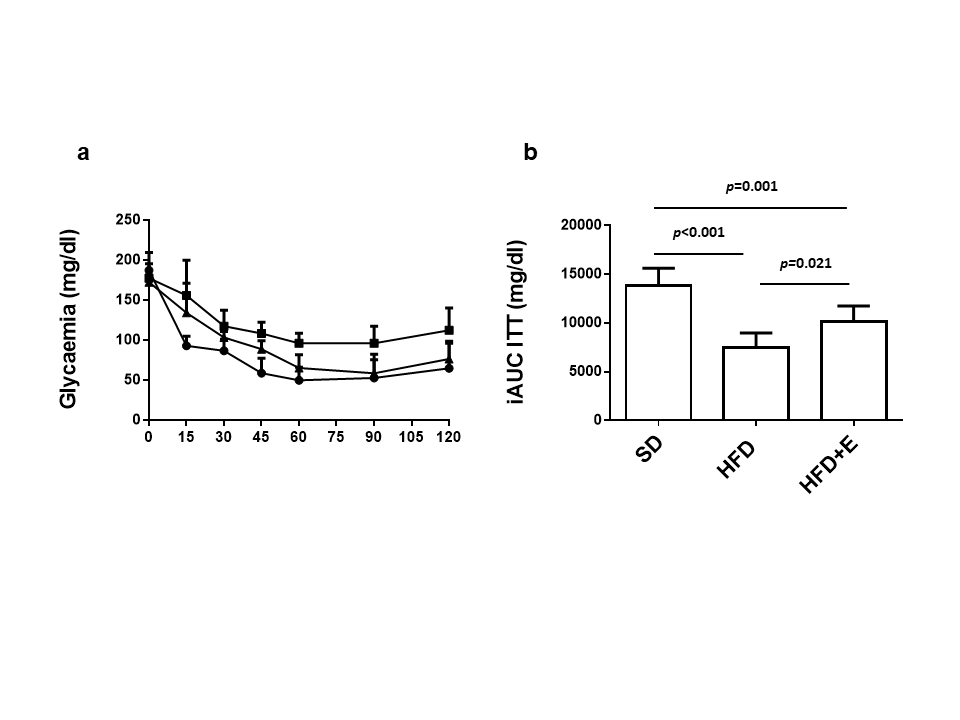

Supplement: Supplementary file 1 [file nutrients-13-01281-s001.zip › nutrients figures/Figure 2 03.04.tif]

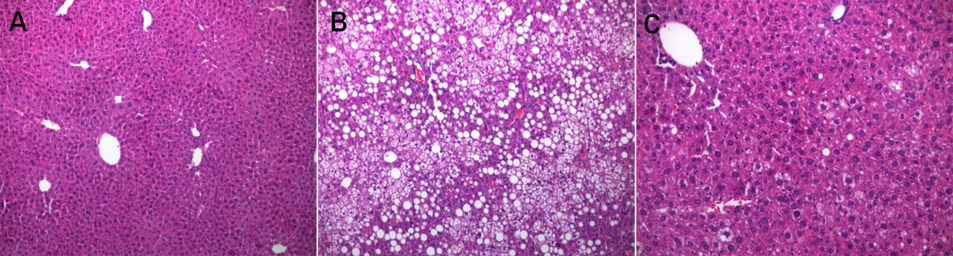

Supplement: Supplementary file 1 [file nutrients-13-01281-s001.zip › nutrients figures/Figure 3.tif]

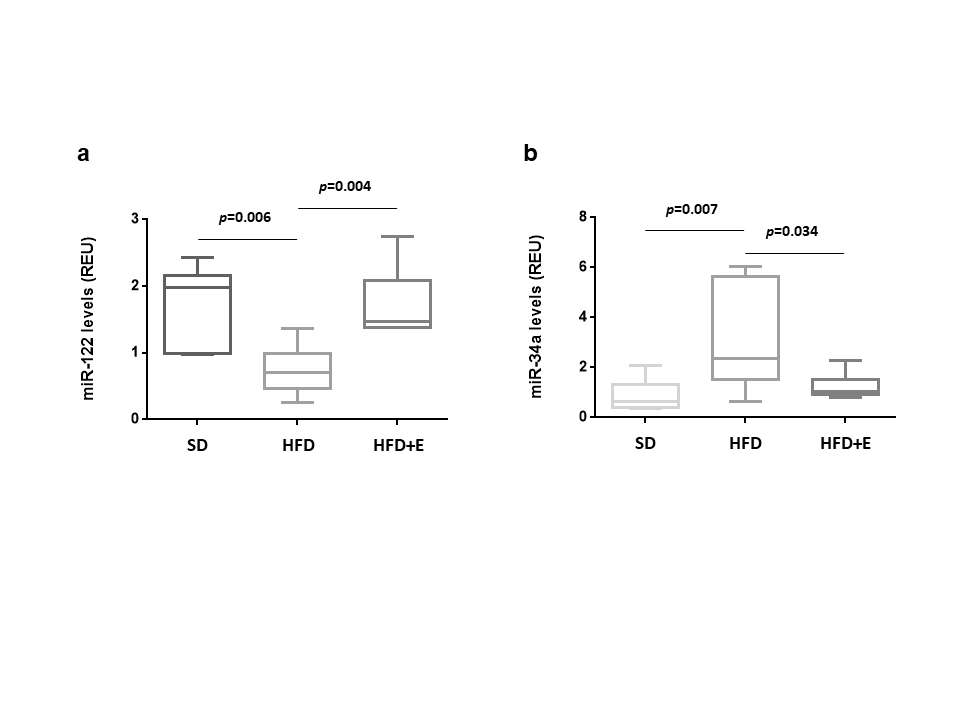

Supplement: Supplementary file 1 [file nutrients-13-01281-s001.zip › nutrients figures/Figure 4 03.04.tif]

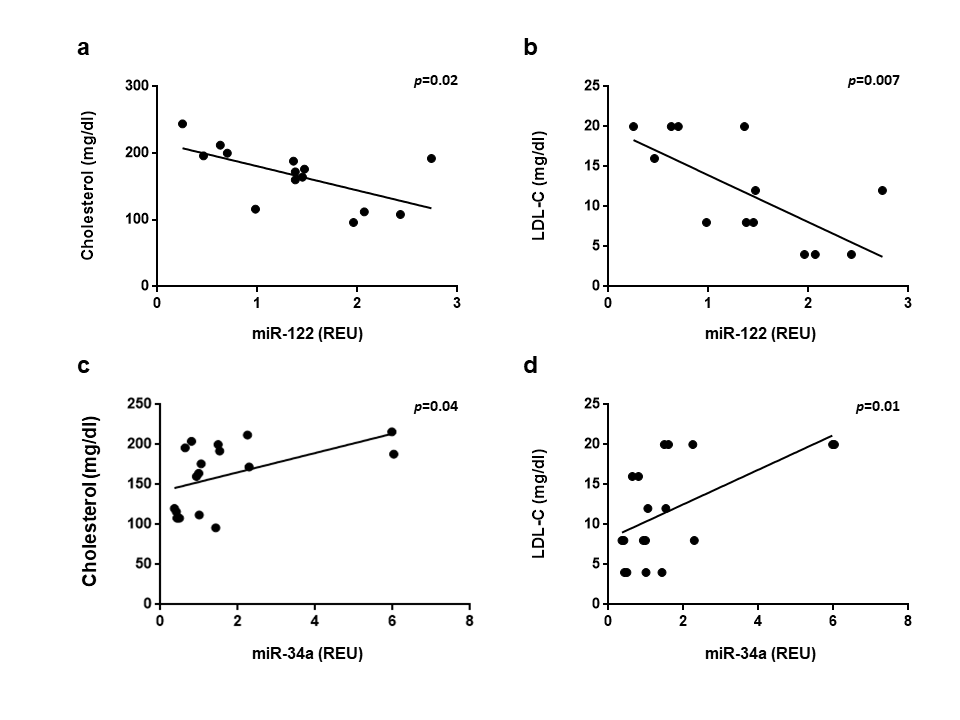

Supplement: Supplementary file 1 [file nutrients-13-01281-s001.zip › nutrients figures/Figure 5_corr.tif]

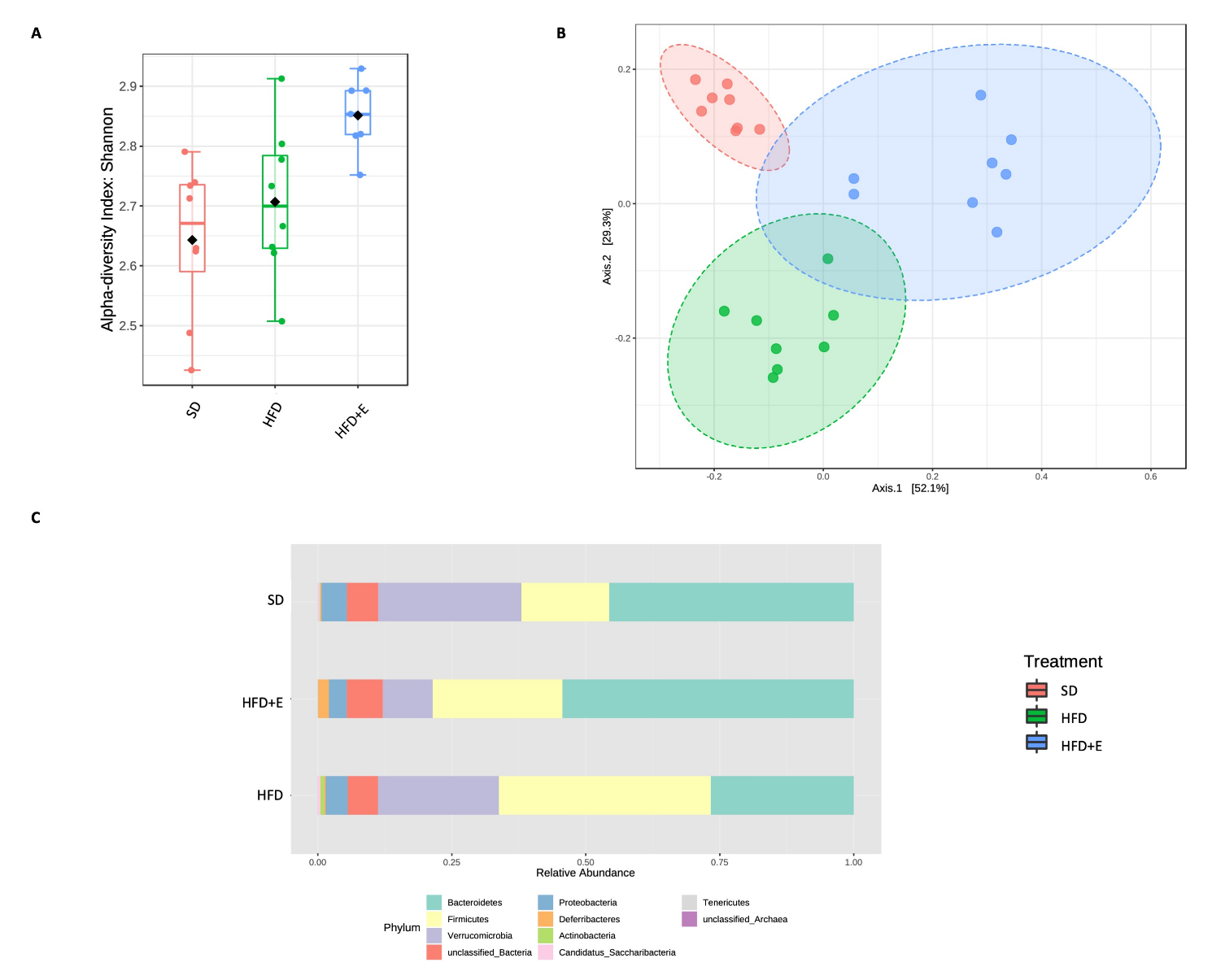

Supplement: Supplementary file 1 [file nutrients-13-01281-s001.zip › nutrients figures/Figure 6.tif]

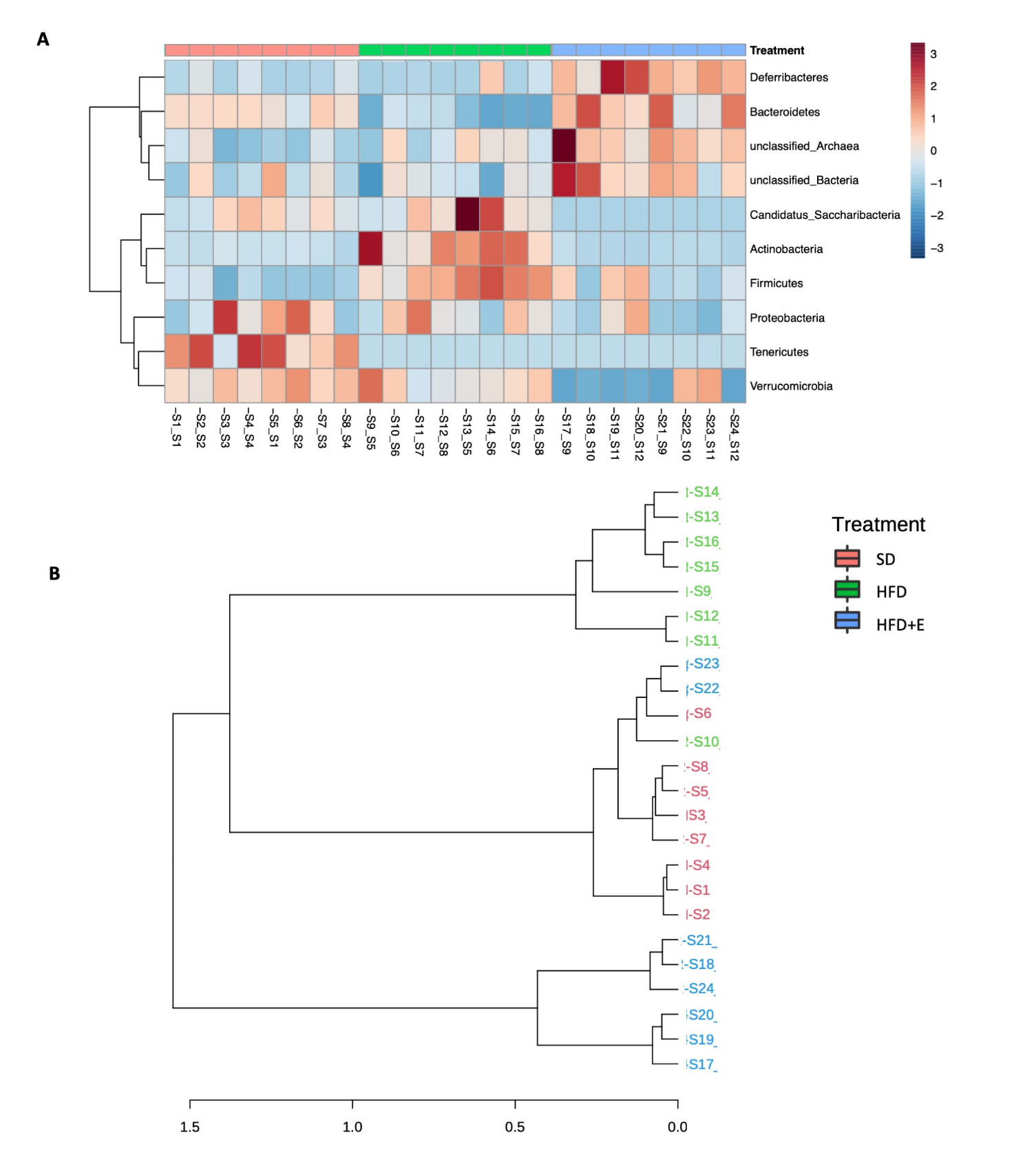

Supplement: Supplementary file 1 [file nutrients-13-01281-s001.zip › nutrients figures/Figure 7.tif]

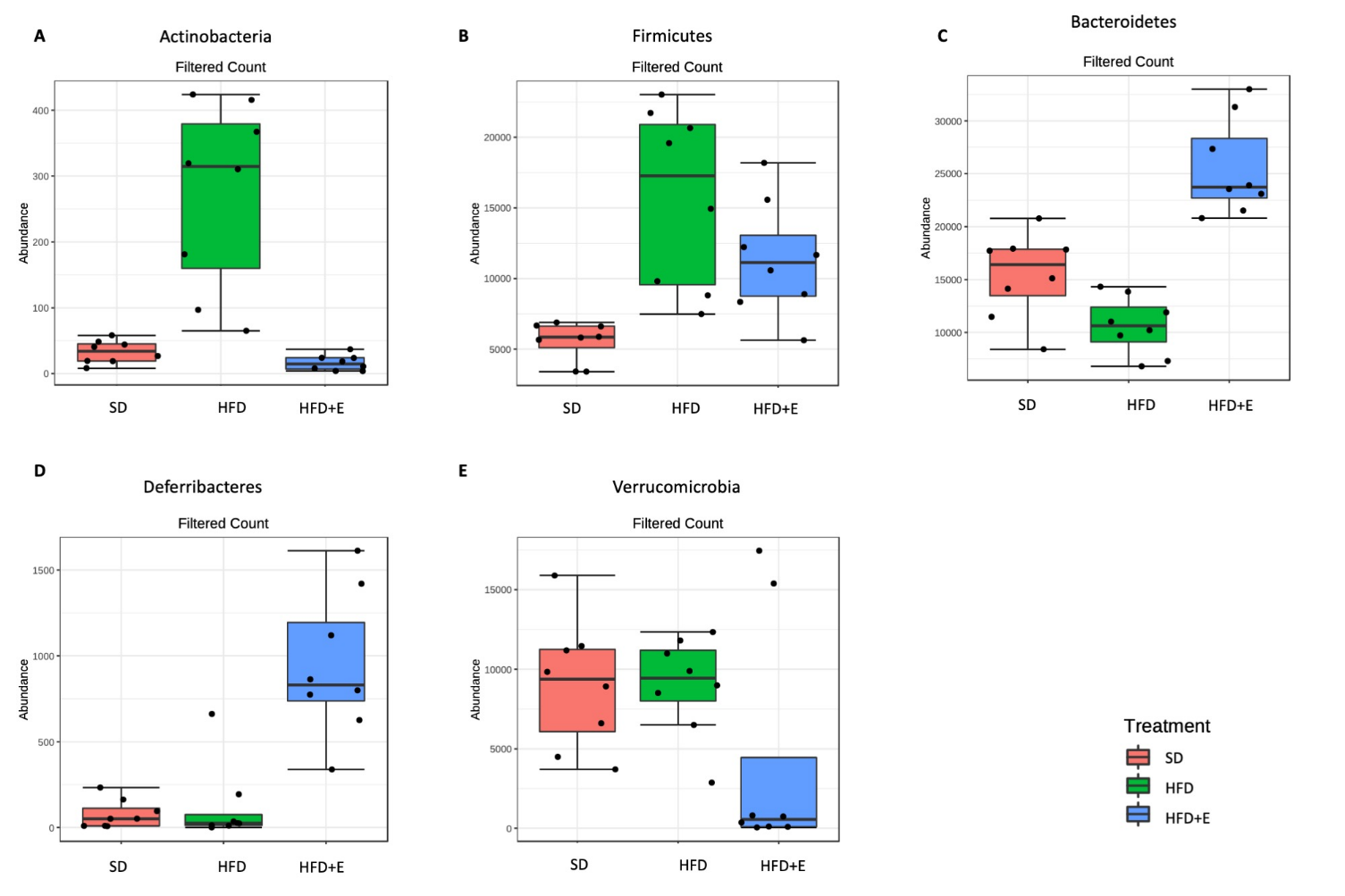

Supplement: Supplementary file 1 [file nutrients-13-01281-s001.zip › nutrients figures/Figure 8.tif]

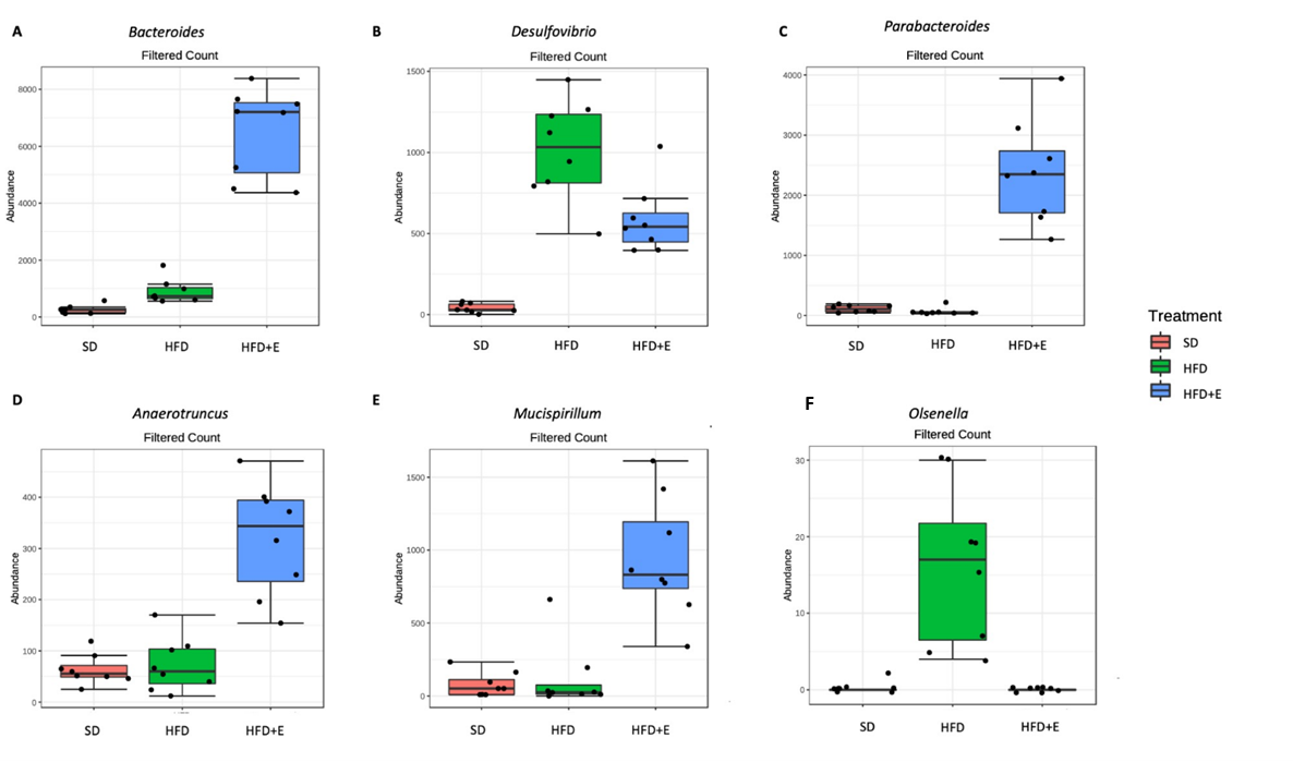

Supplement: Supplementary file 1 [file nutrients-13-01281-s001.zip › nutrients figures/Figure 9.tif]
